# A Replication of a Possible Relationship between Elderly Suicide Rates and Smoking Using Five-Year Data on Suicide Rates. A Cross-National Study

**DOI:** 10.5249/jivr.v2i1.44

**Published:** 2010-01

**Authors:** Ajit Shah

**Affiliations:** ^a^International School for Communities, Rights and Inclusion, University of Central Lancashire, Preston, United Kingdom and West London Mental Health NHS Trust, London, United Kingdom.

## Abstract

**Background:**

There is a paucity of studies examining the relationship between smoking and elderly suicides. A recent cross-national study, using one-year cross-sectional data on suicide rates, reported an absence of an independent relationship between elderly suicide rates and the national prevalence of smoking. National aggregates of suicide rates can randomly fluctuate year on year and may lead to erroneous findings in cross-sectional ecological studies when only data from a single year are utilised.

**Methods:**

The relationship between the national prevalence of smoking and suicide rates in both sexes in the age-bands 65-74 and 75+ years was examined using a one-year average of five years data on suicide rates using data from the World Health Organisation and United Nations Development Programme.

**Results:**

On univariate analysis, the national prevalence of smoking in males was positively correlated with suicide rates in males aged 65-74 and 75+ years, but this relationship was absent in females. On multivariate analysis there was no independent relationship between the national prevalence of smoking in males and suicide rates in males in both the elderly age-bands.

**Conclusions:**

The findings of this study, using a one-year average of five years data on suicide rates and a more recent data set, suggests that the absence of an independent relationship between the national prevalence of smoking and elderly suicide rates was accurate and robust.

## Introduction

Completed suicides were associated with smoking cigarettes in cohort studies of mainly female registered nurses in the United States (US),^1^ both sexes in the Finnish general population,^2^male army recruits in the US,^3^ males in the US general population^4^, Swedish army recruits^5^and males aged 40-69 years in the Japanese general population.

This relationship between completed suicides and smoking was “dose-dependent”.^1-6^ Moreover, it was also maintained in some studies after controlling for confounding variables including demographic characteristics, socio-economic characteristics, levels of alcohol consumption, psychiatric symptoms and physical health,^2,3^ but disappeared in one study.^5^

There has been much debate about the causal link between smoking and completed suicides.^4,7,8^ A number of potential explanations for this relationship have been proposed. First, there may be a causal link between smoking and depressive illness.^1,2^ This may be mediated through the central effects of nicotine^2^. Japanese suicide victims with a history of smoking had higher levels of nicotine and cotinine in their blood and urine.^9,10^ the authors speculated that there may be a marked increase in smoking among habitual smokers before committing suicide. Second, depressed smokers may find it particularly difficult to quit smoking.^1,7^ Third, other factors, including low self esteem^1^ and alcohol and substance misuse,^11^ may predispose to both smoking and suicide. Fourth, depressed patients may have a higher prevalence of smoking.^7^ Fifth, smoking and suicide may be linked to other disorders like cancer:^1,7^ occult carcinomas may clinically present with depressive illness,^12^ and occult carcinomas have been discovered at post mortem in elderly suicide victims.^13^ Finally, smoking may directly enhance suicidal behaviour. There is evidence of serotonergic hypofunction in psychiatric patients with lethal suicidal behaviour,^14^ and an inverse relationship between smoking and indices of serotonin function has been observed in psychiatric patients with depressive illness.^15^

There is a paucity of studies examining the relationship between smoking and elderly suicides. If such a relationship exists then it may have important implications for prevention at a public health level. A cohort study of elderly people in a retirement community in the US also reported an association between completed suicides and smoking.^16^ A recent cross-national study using one-year cross-sectional data on suicide rates also demonstrated this relationship in elderly males.^17^ However, this relationship disappeared in multivariate analysis when other confounding variables including socio-economic status, income inequality, healthcare expenditure, life expectancy and child mortality rates were controlled for.^17^ National aggregates of suicide rates can randomly fluctuate year on year and may lead to erroneous findings in cross-sectional ecological studies when only data from a single year are utilised.^18^ Therefore, a cross-national study was undertaken to replicate the previously observed relationship between elderly suicide rates and smoking by using a one-year average of five-year national aggregate data on elderly suicide rates. The current study also used data on suicide rates more recent than that used in the earlier study.^17^

## Methods

Data on elderly suicide rates for males and females in the age-bands 65-74 years and 75+ years was ascertained from the World Health Organisation (WHO) website (http://www.who.int/whosis/database/mort/table1.cfm).  For a small number of countries only the raw figures for the number of suicides were available from the WHO website. Suicide rates for these countries were calculated by dividing the number of reported suicides by the population size in the relevant age-band and sex group available on the same website. Data on suicide rates were collected for each of the latest five consecutive years for which data were available. The one-year average suicide rate was calculated by dividing the sum of the suicide rates for each of the five years by five. The median (range) for the latest year of the suicide rate data was 2005 (1991-2006);

National-level aggregate data on the prevalence of smoking in males and females was ascertained from the United Nations Development Programme (UNDP) website for the years 2002-2004(http://hdr.undp.org/en/media/HDI_ 2008_EN_Tables.pdf).

Univariate analysis, using Pearson’s correlation coefficient (rho), were initially conducted to examine the relationship between the national prevalence of smoking and suicide rates in both sexes in both the elderly age-bands.

In individual-level cohort and case-control studies several confounding variables, as discussed in the Introduction, may influence the relationship between smoking and suicide. Also, in cross-national studies a number of variables at national aggregate-level were associated with elderly suicide rates.^19-22^ Therefore, to ascertain the independent relationship between the prevalence of smoking and suicide rates with national-level aggregate data, multiple linear regression analysis with the Enter method was undertaken for any of the four groups of suicide rates if a significant relationship between smoking and suicide rates was observed on univariate analysis. The independent variable was the suicide rate. The national prevalence of smoking in males or females and previously reported correlates of elderly suicide rates were used as the dependent variables to replicate the findings of an earlier similar study,^17^ and included: (i) the proportion of elderly in the total population; (ii) male or female child mortality rates; (iii) male or female life expectancy; (iv) per capita gross national domestic product (GDP) - a measure of socio-economic status; (v) percentage of GDP spent on health; (vi) per capita health expenditure; and, (vii) the Gini coefficient - a measure of income inequality.

The WHO website (www.who.int/countries/en/) provided data on the proportion of people over the age of 60 years in the general population, males and female child mortality rates, male and female life expectancy, the GDP, the proportion of GDP spent on health and per capita expenditure on health for the year 2006. The UNDP website (http://hdr.undp.org/en/media/HDI_2008_EN_Tables.pdf)  provided data on the Gini coefficient. Data on the Gini coefficient were collected for the latest available year and the median (range) of this latest year across the different countries was 2000 (1990-2003).

## Results

Data on both elderly suicide rates and the national prevalence of smoking were available for 38 countries. The national prevalence of smoking in males was significantly correlated with suicide rates in males aged 65-74 years (r=+0.62, P<0.00001) and males aged 75+ years (r=+0.47, P=0.003). There was no significant relationship between the national prevalence of smoking in females and suicide rates in females in either of the elderly age-bands.

Multivariate analyses were only conducted for male suicide rates as there was no significant correlation between female suicide rates and the national prevalence of smoking in females. Data on elderly suicide rates, the national prevalence of smoking in males, the proportion of elderly in the general population, male child mortality rates, male life expectancy, per capita GDP, the Gini coefficient, the proportion of GDP spent on health, and per capita expenditure on health were available for 33 countries. Tables 1 and 2 provides more detailed information on the character-istics of multiple regression analysis for males aged 65-74 years and 75+ years respectively. Suicide rates in males aged 65-74 years were independently predicted by male child mortality rates (P=0.002) and male life expectancy (P=0.002). Suicide rates in males aged 75+ years were independently predicted only by male child mortality rates (P=0.046). The national prevalence of smoking in males did not independently predict suicide rates in males aged 65-74 years and males aged 75+ years.

**Table:** Table 1: **Characteristics of multiple regression analysis for males aged 65-74 years**

Model	Unstandardized Coefficients		Standardized Coefficients	t	p	Correlations			Collinearity Statistics	
	B	Std. Error	Beta			Zero-order	Partial	Part	Tolerance	VIF
Constant	199.600	48.768		4.093	.000					
Giniindex #	-.373	.331	-.154	-1.128	.264	-.325	-.148	-.112	.527	1.898
Propopover60¥	.687	.744	.181	.924	.360	.422	.121	.091	.255	3.926
Gross national domestic product	.000	.000	-.389	-1.390	.170	.073	-.181	-.138	.125	7.999
Per capita health expenditure	.006	.005	.388	1.263	.212	.071	.165	.125	.104	9.636
Percentage GDP* spent on health	-2.712	1.762	-.256	-1.539	.129	-.021	-.200	-.152	.353	2.834
Male life expectancy	-1.801	.600	-.508	-3.004	.004	-.052	-.370	-.297	.343	2.920
Male child mortality	-.887	.227	-.713	-3.915	.000	-.398	-.460	-.388	.296	3.382

#Giniindex = A measure of income inequality ¥Propopover60= Proportion of population over the age of 60 years *GDP = Gross Nattional Domestic Product P=probability T=t-value

**Table:** Table 2: **Characteristics of multiple regression analysis for males aged 75+ years**

Model	Unstandardized Coefficients		Standardized Coefficients	t	p	Correlations			Collinearity Statistics	
	B	Std. Error	Beta			Zero-order	Partial	Part	Tolerance	VIF
Constant	170.774	74.871		2.281	.026					
Giniindex #	-.576	.508	-.166	-1.133	.262	-.338	-.148	-.121	.527	1.898
Propopover60¥	.905	1.143	.167	.792	.432	.492	.104	.084	.255	3.926
Gross national domestic product	.000	.001	-.155	-.514	.609	.235	-.068	-.055	.125	7.999
Per capita health expenditure	.002	.008	.088	.267	.790	.214	.035	.028	.104	9.636
Percentage GDP* spent on health	-.749	2.706	-.050	-.277	.783	.137	-.037	-.029	.353	2.834
Male life expectancy	-1.324	.921	-.262	-1.438	.156	.158	-.187	-.153	.343	2.920
Male child mortality	-1.024	.348	-.577	-2.943	.005	-.487	-.363	-.313	.296	3.382

#Giniindex = A measure of income inequality ¥Propopover60= Proportion of population over the age of 60 years *GDP = Gross Nattional Domestic Product P=probability T=t-value

## Discussion

Some methodological issues need consideration. National-level aggregate data on suicide rates should be viewed cautiously because: data were not available from all countries;^23,24^ the validity of this data was unclear;^24,25^ the legal criteria for the proof of suicide vary between countries and in different regions within a country;^24,26^ some countries, particularly low-income countries, may have poor death registration facilities;^26^  and, cultural and religious factors and stigma attached to suicide may lead to under-reporting of suicides.^24,27^  Some of these methodological problems were minimized by using one-year average of five year data on suicide rates. National-level aggregate data on socio-economic status and health-related measures should also be viewed cautiously because:^22^ the validity of this data is unclear; some countries may have poor registration facilities for data on health-related measures; some countries may have poor infrastructure for providing accurate financial data; and, their influence on social distribution of risk factors may be complex.^28^ National-level aggregate data on the prevalence of smoking should also be viewed with caution because data were only available for 38 countries and the validity of this data was unclear. Given the large number of independent variables in the regression analysis only 38 data points may have been too small and thus make the model unstable. The data on smoking was only for one year (the latest available year) because data for five consecutive years are not available. Also, it was not possible to examine the relationship between the prevalence of smoking in the years preceding the years of suicide data as such data were not available for all countries, although the median year of data for suicide was earlier than the median year for the latest year of suicide rate data. Nevertheless, the latest and best available data set were used.

The absence of an independent relationship between the national prevalence of smoking in males and females and suicide rates in both sexes is both the elderly age-bands is not consistent with the relationship between smoking and completed suicides in cohort and case-control studies at an individual-level in younger subjects (as described in the Introduction) and a cohort study of elderly people in a retirement community in the US.^16^ There may be several possible explanations for this. First, the methodological issues described above may account for the negative finding. Second, national-level aggregate data may not capture the direct effect of smoking on suicide at an individual-level, particularly when data were only available for 38 countries. Third, the absence of a relationship may be genuine. This is consistent with an absence of a relationship between suicidal ideation and smoking in elderly general practice attenders in Australia^29^ and completed suicide in Swedish army when confounding variables were controlled.^5^ Fourth, the discrepancy between univariate and multivariate analysis may be because of outliers and skewness of the data. Fifthly, the high VIF values for collineality and the partial correlation values suggest collineality between the independent variables; this is likely to be an issue also with partial least square regression. Additionally, the findings were also consistent with an almost identical cross-national study which used only one-year cross-sectional data on elderly suicide rates.^17^ The replication of the findings of the latter study by using more recent data on suicide rates and a one-year average of five years data on suicide rates (to minimize the effect of year on year random fluctuation in suicide rates) suggests that the original findings were accurate and robust, but they are in the context of the methodological issues described above. It may worth conducting individual level studies in a small number of countries with varying socio-economic status, health status and income inequality; this was beyond the scope of this study.
